# Draft Genome Sequence of Type Strain *Clostridium pasteurianum* DSM 525 (ATCC 6013), a Promising Producer of Chemicals and Fuels

**DOI:** 10.1128/genomeA.00232-12

**Published:** 2013-02-21

**Authors:** Sugima Rappert, Lifu Song, Wael Sabra, Wei Wang, An-Ping Zeng

**Affiliations:** Institute of Bioprocess and Biosystems Engineering, Hamburg University of Technology, Hamburg, Germany

## Abstract

*Clostridium pasteurianum*, an anaerobic bacterium able to utilize atmospheric free nitrogen for biosynthesis, has recently been proven to be a promising producer of chemicals and fuels, such as 1,3-propanediol and *n*-butanol. Here, we report the high-quality draft genome sequence of DSM 525, a type strain of *C. pasteurianum*.

## GENOME ANNOUNCEMENT

*Clostridium pasteurianum* is an anaerobic bacterium that can utilize free nitrogen from the atmosphere for biosynthesis. The nitrogen-fixing mechanism of *C. pasteurianum* has been widely investigated since the 1950s (1–9). The production of *n*-butanol (10–15) and 1,3-propanediol (11, 13, 16–18) using strains of *C. pasteurianum* has been studied mainly in recent years, showing promising potential applications for *C. pasteurianum* in industrial biotechnology.

Here, we present the first draft genome sequence of the *C. pasteurianum* type strain DSM 525, obtained using the high-throughput Illumina sequencing technology, which was performed by the Beijing Genomics Institute (BGI) in Shenzhen, China, with a paired-end library. The sequence reads were assembled with Short Oligonucleotide Analysis Package 2 (SOAP2) (19) with a k-mer setting of 63, which was determined by the optimal assembly result. The assembled genome sequence was annotated using the NCBI Prokaryotic Genomes Automatic Annotation Pipeline (PGAAP) (http://www.ncbi.nlm.nih.gov/genomes/static/Pipeline.html). Open reading frames (ORFs) were predicted with Glimmer (20), GeneMark (21), and Prodigal (22); tRNAs were predicted using tRNAscan-SE (23); and ribosomal RNAs were predicted by sequence similarity search using BLAST against an RNA sequence database and/or using Infernal and Rfam models. The G+C content was calculated using the genome sequence. Prophage analysis was performed by a BLAST search against the prophage protein database derived from the prediction results of the Prophinder software and A Classification of Mobile Genetic Elements (ACLAME) database (http://aclame.ulb.ac.be/Tools/Prophinder/).

The final genome sequence of the strain DSM 525 comprises 4,285,687 bases, which are assembled into 37 contigs. It has a G+C content of 29.75%. In total, 3,964 genes were predicted using the PGAAP, including 3,887 protein-coding sequences (CDSs), 74 tRNAs, and 3 rRNA genes. According to the annotation, *C. pasteurianum* is predicted to possess a complete glycolysis pathway, while the tricarboxylic acid cycle and the pentose phosphate pathways are incomplete. Of special importance is the ability of this bacterium to produce *n*-butanol and 1,3-propanediol as major byproducts of glycerol in a minimal medium with low concentration of yeast extract (1 g/liter). Interestingly, unlike the traditional acetone-butanol-ethanol fermentation, acetone is not a byproduct of glycerol fermentation. No prophage elements were detected.

### Nucleotide sequence accession numbers.

This Whole Genome Shotgun project has been deposited at DDBJ/EMBL/GenBank under the accession no. ANZB00000000. The version described in this paper is the first version, ANZB01000000.
